# Deep Learning for Prediction of Progression and Recurrence in Nonfunctioning Pituitary Macroadenomas: Combination of Clinical and MRI Features

**DOI:** 10.3389/fonc.2022.813806

**Published:** 2022-04-20

**Authors:** Yan-Jen Chen, Hsun-Ping Hsieh, Kuo-Chuan Hung, Yun-Ju Shih, Sher-Wei Lim, Yu-Ting Kuo, Jeon-Hor Chen, Ching-Chung Ko

**Affiliations:** ^1^ Department of Electrical Engineering, National Cheng Kung University, Tainan, Taiwan; ^2^ Graduate Institute of Electronics Engineering, National Taiwan University, Taipei, Taiwan; ^3^ Department of Anesthesiology, Chi Mei Medical Center, Tainan, Taiwan; ^4^ Department of Hospital and Health Care Administration, College of Recreation and Health Management, Chia Nan University of Pharmacy and Science, Tainan, Taiwan; ^5^ Department of Medical Imaging, Chi-Mei Medical Center, Tainan, Taiwan; ^6^ Department of Neurosurgery, Chi Mei Medical Center, Tainan, Taiwan; ^7^ Department of Nursing, Min-Hwei College of Health Care Management, Tainan, Taiwan; ^8^ Department of Medical Imaging, Kaohsiung Medical University Hospital, Kaohsiung, Taiwan; ^9^ Department of Radiological Sciences, University of California, Irvine, Irvine, CA, United States; ^10^ Department of Radiology, E-DA Hospital, I-Shou University, Kaohsiung, Taiwan; ^11^ Department of Health and Nutrition, Chia Nan University of Pharmacy and Science, Tainan, Taiwan; ^12^ Institute of Biomedical Sciences, National Sun Yat-Sen University, Kaohsiung, Taiwan

**Keywords:** deep learning, pituitary, macroadenoma, progression, recurrence, MRI, MLP, CNN

## Abstract

**Objectives:**

A subset of non-functioning pituitary macroadenomas (NFMAs) may exhibit early progression/recurrence (P/R) after tumor resection. The purpose of this study was to apply deep learning (DL) algorithms for prediction of P/R in NFMAs.

**Methods:**

From June 2009 to December 2019, 78 patients diagnosed with pathologically confirmed NFMAs, and who had undergone complete preoperative MRI and postoperative MRI follow-up for more than one year, were included. DL classifiers including multi-layer perceptron (MLP) and convolutional neural network (CNN) were used to build predictive models. Categorical and continuous clinical data were fed into the MLP model, and images of preoperative MRI (T2WI and contrast enhanced T1WI) were analyzed by the CNN model. MLP, CNN and multimodal CNN-MLP architectures were performed to predict P/R in NFMAs.

**Results:**

Forty-two (42/78, 53.8%) patients exhibited P/R after surgery. The median follow-up time was 42 months, and the median time to P/R was 25 months. As compared with CNN using MRI (accuracy 83%, precision 87%, and AUC 0.84) or MLP using clinical data (accuracy 73%, precision 73%, and AUC 0.73) alone, the multimodal CNN-MLP model using both clinical and MRI features showed the best performance for prediction of P/R in NFMAs, with accuracy 83%, precision 90%, and AUC 0.85.

**Conclusions:**

DL architecture incorporating clinical and MRI features performs well to predict P/R in NFMAs. Pending more studies to support the findings, the results of this study may provide valuable information for NFMAs treatment planning.

## Introduction

Pituitary adenomas constitute up to 15% of all intracranial tumors (1), and the majority of these tumors are nonfunctioning adenomas (2, 3). Nonfunctioning pituitary macroadenomas (NFMAs), defined as a tumor larger than 10 mm in diameter, are the most common presentation among pituitary tumors (2, 3). Clinically, NFMAs often cause bitemporal hemianopia due to compression of the optic chiasm. Endocrine dysfunction such as hypopituitarism is found in some patients because of tumor compression of the normal pituitary gland. According to 2017 WHO classification system, pituitary tumors are classified as adenoma, carcinoma, or blastoma (4). Although most NFMAs are diagnosed as benign adenomas, up to 52.7% of these tumors may undergo early progression/recurrence (P/R) after surgical resection (5). The trans-sphenoidal approach (TSA) is the optimal surgery for NFMAs in current clinical practice. However, gross total resection (GTR) is often difficult to achieve for large solid NFMAs with extrasellar extension (6). Although postoperative adjuvant radiotherapy (RT) can be used to reduce P/R in NFMAs after surgery, this method may result in irreversible pituitary insufficiency and other long-term complications (7).

Conventional MRI features such as cavernous sinus invasion, extrasellar extension, and absence of tumor apoplexy have been reported as significant imaging parameters related to P/R in NFMAs (8–11). However, most of these parameters are qualitative and subjective with inter-observer variation. Currently, machine learning (ML) algorithms have become a popular tool in cancer prognosis and prediction because it offers quantitative and objective information (12). Integration of mixed data such as clinical data and diagnostic imaging is an obvious trend toward personalized medicine (13). Imaging-based ML algorithms include two popular methods: handcrafted feature-based and automatic feature-learning based models (14). For automatic feature-learning based models, deep learning (DL) is a powerful method for building predictive models for cancer diagnosis (15). Both multilayer perceptron (MLP) and convolutional neural network (CNN) are popular DL models and can be used for image classification. As compared with MLP that takes vector as input, CNN takes tensor as input and better understands spatial relations between pixels of images. Thus, CNN performs better than MLP for complicated images and videos classification (16). CNN had attracted attention when large-scale CNN for image classification successfully outperformed all other techniques in the ImageNet 2012 competition (17). CNN is designed to learn spatial hierarchies of features automatically and adaptively through backpropagation by using three building blocks: convolution layers, pooling layers, and fully connected layers. Recently, several studies have reported that deep CNN-based approaches can achieve state-of-the-art performance in lesion detection and cancer diagnosis (18–21).

Regarding clinical applications in the management of pituitary adenomas, DL models such as MLP or CNN have been used to evaluate tumor secreting function (22), tumor consistency (23, 24), detection of pituitary adenoma (25, 26), classification of sellar tumor types (27), and predicting the extent of surgery (28). U-Net and derived DL models are currently considered as optimal for image segmentation (29). Recently, DL showed high accuracy in predicting suboptimal postoperative outcomes in functional pituitary adenomas (30). However, the DL gmodels for predicting tumor recurrence in NFMAs have not yet been reported. The purpose of this study was to investigate the roles of DL in predicting P/R in NFMAs, using the combination of clinical and MRI features in MLP and CNN architectures.

## Materials and Methods

### Ethics Statement

The study was approved by the Institutional Review Board (IRB no. 10902-009) of our center. Signed informed consent was waived because the retrospective nature of this study does not affect the healthcare of the included patients. All patients’ medical records and imaging data were de-identified before analysis.

### Patient Selection

The inclusion criteria of this study were patients diagnosed with benign NFMAs by brain MRI (diameter > 10 mm) and pathological confirmation. All included patients must have undergone complete preoperative brain MRI, at least one postoperative MRI performed at 3 to 6 months after surgery, and serial postoperative brain MRI follow-up for more than 1 year. Patients with evidence of hormone hypersecretion in clinical, biochemical, and histopathological examinations were excluded. Based on data of previous studies (8, 31), prolactinoma is considered unlikely if the prolactin levels are below 100 ng/mL, and this diagnosis was thereafter excluded by immunocytochemical tests. Patients who received adjuvant RT before P/R were also excluded. From June 2009 to December 2019, 78 patients (49 men, 29 women, age 18 - 80 years; median age, 53.5 years) were included in this study according to above-mentioned inclusion and exclusion criteria. Total 42 P/R patients and 36 non-P/R patients were included. Seventy-six patients underwent surgery performed by TSA, and 2 patients received TSA and craniotomy due to large size tumors (tumor diameters of 6.5 cm and 6.1cm). The mean follow-up time for all patients was 42 months (range, 12 to 115 months). In 42 patients with P/R, the mean time to P/R was 25 months (range, 6 to 68 months).

### Image Acquisition

The MR images were acquired using a 1.5-T (Siemens, MAGNETOM Avanto) (n = 39), 1.5-T (GE Healthcare, Signa HDxt) (n = 23), or a 3-T (GE Healthcare, Discovery MR750) (n = 16) MR scanner equipped with 8-channel head coils in each machine. The analyzed MR images included coronal T2-weighted image (T2WI) and coronal contrast-enhanced (CE) T1-weighted image (T1WI). CE T1WI images were obtained with intravenous administration of 0.1 mmol/kg of body weight of gadobutrol (Gadovist) or gadoterate meglumine (Dotarem). Detailed MR imaging parameters were described in **Supplementary File 1**.

### Clinical and Radiological Variables

The clinical data were obtained from patients’ medical records. A neuroradiologist (C.C.K, with 11 years of experience in radiology) and a neurosurgeon (S.W.L, with 15 years of experience in neurosurgery) evaluated preoperative clinical and radiological features on the Picture Archiving and Communication System (PACS) (INFINITT Healthcare, Seoul, Korea) workstations (summarized in **Table 1**). For equivocal cases, judgment was made by consensus. Evaluation of cavernous sinus invasion (Knosp classification) (32) and extrasellar extension (Hardy’s classification) (33) were determined on preoperative coronal T2WI and CE T1WI. Quantitative MRI features were measured on coronal CE T1WI.

**Table 1:** The clinical data and MR features of nonfunctioning pituitary macroadenomas (NFMAs) with and without progression/recurrence (P/R).

	P/R	Non-P/R	*p*
**Number of patients**	42	36	
**Sex**			0.089
Male	30 (71.4%)	19 (52.8%)	
Female	12 (28.6%)	17 (47.2%)	
**Age (y)**	56 (46, 66)	49 (33.5, 64.5)	0.166
**Body mass index (BMI)**	24.8 (23.3, 26.3)	24.5 (22, 27)	0.452
**Clinical symptoms**			
Visual disturbance	39 (92.9%)	20 (55.6%)	<0.001*
Headache	12 (28.6%)	17 (47.2%)	0.089
Decreased libido, sexual dysfunction, and/or amenorrhea/oligomenorrhea	5 (11.9%)	2 (5.6%)	0.442
Incidental	2 (4.8%)	7 (19.4%)	0.073
**Hypopituitarism**			0.033*
No	22 (52.4%)	29 (80.6%)	
Single	11 (26.2%)	4 (11.1%)	
Multiple	9 (21.4%)	3 (8.3%)	
**Hyperprolactinemia**	12 (28.6%)	9 (25%)	0.723
**Extent of surgical resection**			<0.001*
Gross-total resection (GTR)	3 (7.1%)	16 (44.4%)	
Gross-total resection (STR)	39 (92.9%)	20 (55.6%)	
**Successful chiasmatic decompression**	16 (38.1%)	25 (69.4%)	0.006*
**Cavernous sinus invasion** (Knosp classification Grade 3-4)	13 (31%)	4 (11.1%)	0.034*
**Extrasellar extension** (Hardy’s classification Grade 3-4)	15 (35.7%)	4 (11.1%)	0.012*
**Compression of optic chiasm**	39 (92.9%)	26 (72.2%)	0.015*
**Compression of the 3rd ventricle**	29 (69%)	13 (36.1%)	0.004*
**Hydrocephalus**	3 (7.1%)	1 (2.8%)	0.620
**Giant (> 40 mm)**	12 (28.6%)	2 (5.6%)	0.008*
**Maximum tumor height (mm)**	33 (24, 42)	19 (13.5, 24.5)	<0.001*
**Tumor volume (cm^3^)**	11.9 (4.6, 19.2)	2.7 (1.5, 6)	<0.001*
**Follow-up time (months)**	49.7 (40.4, 59.1)	32 (25, 39.1)	0.005*

Continuous variables were presented as median and interquartile range (IQR).

*Statistical difference (p < 0.05).

### Definitions of Extent of Resection (EOR) and Progression/Recurrence (P/R)

The extent of resection (EOR) was determined by review of preoperative and postoperative MRIs by a neuroradiologist (C.C.K) and a neurosurgeon (S.W.L). According to previously published studies (10, 34, 35), GTR was defined as NFMAs with a residual tumor volume of less than 10% as compared with its original tumor size. In contrast, subtotal resection (STR) was defined as the presence of residual tumor more than 10% of its original volume. For determining P/R in NFMAs, preoperative and serial postoperative MRIs were evaluated by a neuroradiologist (C.C.K) and a neurosurgeon (S.W.L), both of whom were blinded to the clinical outcomes of the studied patients. P/R was defined as progression (enlargement) of the residual tumor after STR or tumor recurrence (regrowth) after GTR observed on serial postoperative MRI (CE T1WI) as compared with the MRI performed at 3 to 6 months after surgery. The threshold of P/R in NFMAs was defined as a more than 2mm increase of residual tumor size in at least one dimension when compared with postoperative serial MRIs on CE T1WI (8, 10, 11, 35). For the determination of P/R, the inter-observer reliability with Cohen k value of 0.9 was obtained. Judgment was made by consensus in equivocal cases. Several studies showed the median time to early P/R in NFMAs was within 30 months (10, 36, 37), and the median follow-up time in the present study (both P/R and non-P/R groups) was longer than this interval.

### Image Pre-Processing

**Figure 1:**
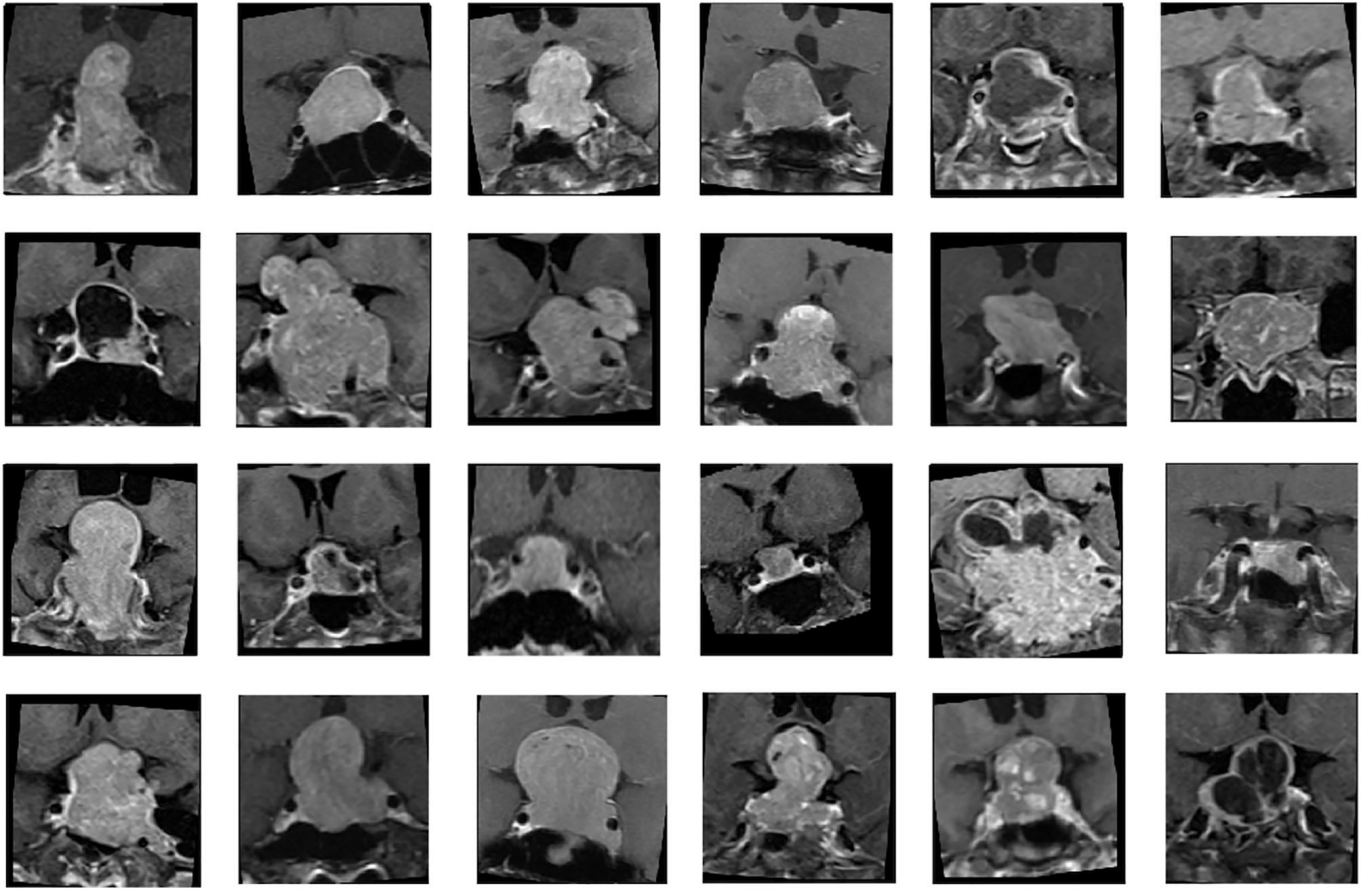
Samples of nonfunctioning pituitary macroadenomas (NFMAs) on coronal contrast-enhanced (CE) T1WI analyzed in CNN models.

Two MRI sequences, coronal T2WI and coronal CE T1WI, were used for analysis (**Figure 1**). Image pre-processing was performed for all MRI images in the training and validation datasets. Python image-processing package (pydicom) (38) was applied to MRI dicom files to obtain pixel data. Rescaling grey scale between 0 to 255 was performed. To fully exploit the information of tumor tissues, an experienced neuroradiologist (C.C.K) selected one coronal CE T1WI slice showing the largest tumor height as the input image. To allow the neural network model to focus on analyzing the tumor tissue without too much noise, the tumor tissue was moved to the center of the image and the outer region of the tumor image was removed. For each selected image, a cropping region with width/length of one third of the original image size is created. Then, the tumor tissue is placed at the center of this cropping region. The dataset was split into 5 folds for cross-validation. Data augmentations, including random flip, random rotate, random scale, and random shift, were applied to each MR image to enhance the training effectiveness and prevent overfitting (27). Some samples of processed images are shown in the **Figure 1**.

### Architectures of CNN, MLP, and multimodal CNN-MLP

**Figure 2:**
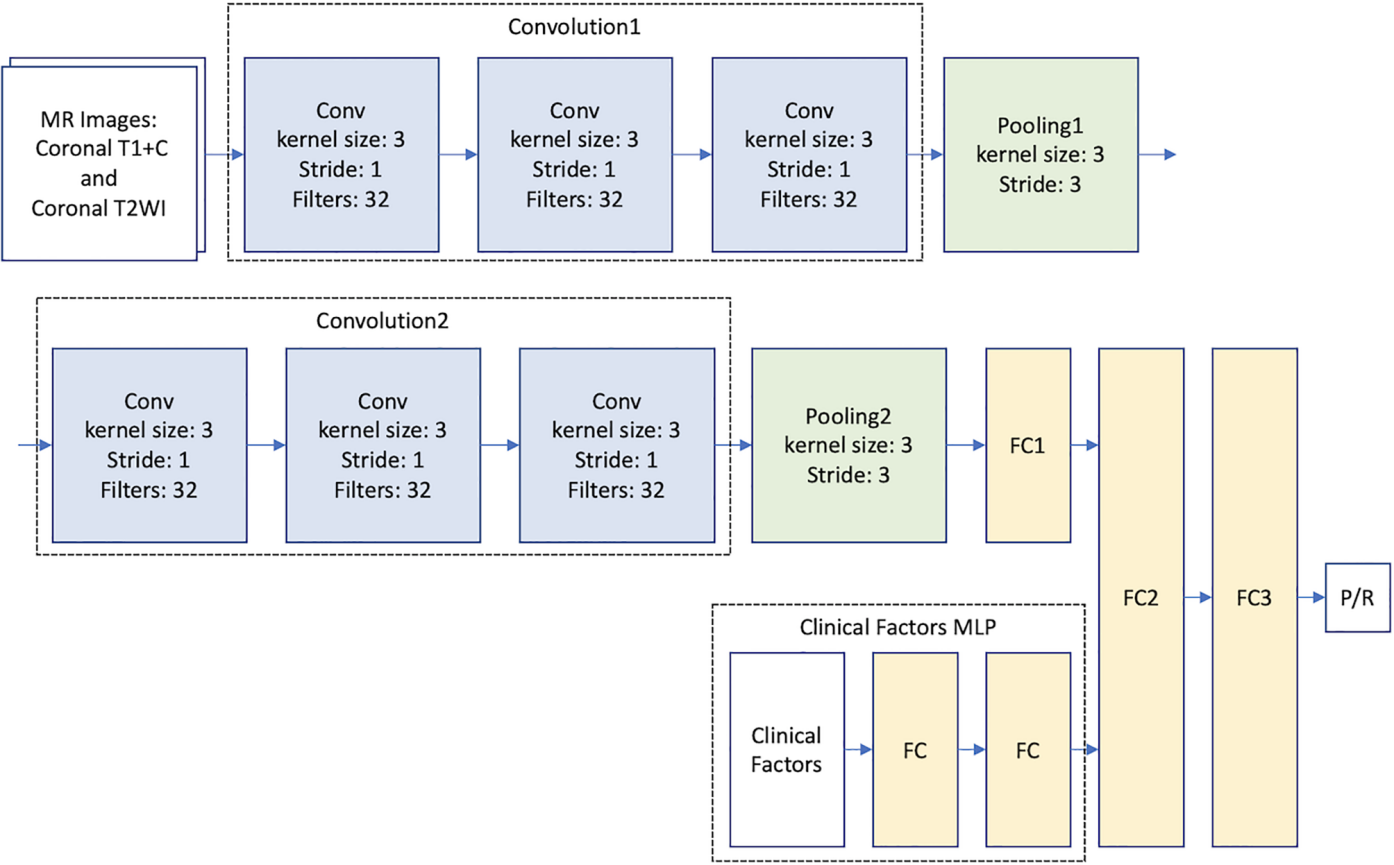
Multimodal CNN_v1-MLP architecture for prediction of progression/recurrence (P/R) in NFMAs.

Because of the small amount of data in this study, modern CNN-based architectures such as AlexNet (17) and GoogleNet (39) cannot be directly applied to train accurate models. Therefore, we proposed to build a relatively light model based on two classical CNN architectures: LeNet (40) and VGG16 (41) (**Figure 2**). For imaging analysis in CNN, our model takes MR images as input, and different imaging sequences (T2WI and CE T1WI) were stacked on the channel axis. This setting gave our model a chance to discover local image features from different MRI sequences. The two convolution layers in LeNet are replaced by convolution blocks from VGG16 (i.e., Convolution 1 and Convolution 2), which are formed by three 3 x 3 convolution layers (**Figure 2**). Then, the extracted image feature from second pooling layer (Pooling 2) is fed to three fully connected (FC) layers (FC1, FC2, and FC3) to predict the P/R. The idea of combining two such CNN models improves the predictive effectiveness. The reason is twofold. First, the original VGG16 is a complex and heavy model that suffers from the lack of data; thus, we set the basic CNN model as LeNet. Second, the convolution block of VGG16 can capture much more multi-scale image features than the original convolution block of LeNet. In this study, the designed architecture improved the predictive effectiveness as compared with applying LeNet or VGG16 individually.

To provide clinical variables (summarized in **Table 1**) to the model, a MLP network that takes clinical factors as input was added before the second fully connected layer (FC2). MLP is a class of neural network, which is good at learning relationships from categorical features. The multimodal CNN-MLP model captures both image and numerical clinical features. The following clinical variables were included in the MLP model: sex, age, body mass index (BMI), clinical symptoms, hypopituitarism, hyperprolactinemia, EOR, chiasmatic decompression, Knosp and Hardy classifications, compression of optic chiasm and 3^rd^ ventricle, hydrocephalus, tumor diameter, and tumor volume (**Table 1**). Details of the multimodal CNN-MLP architecture is shown in **Figure 2**. Another multimodal CNN_v2-MLP model were described in **Supplementary File 2**.

### Training Process

All experiments were trained on one NVIDIA GTX1080ti graphic card with TensorFlow 2.1. We train each model from scratch with the following setting and hyperparameters. All variables were initialized with Glorot uniform (or called Xavier uniform), and Adam optimizer was used. Learning rate initialized at 0.0001 and started decade after 20 epochs. Binary cross entropy was used as the loss function since the final prediction is only progression or recurrence. Each experiment was conducted with 5-fold cross validation to observe the stability and reliability of our model. All P/R and non-P/R case were separated evenly into 5 folds in order to prevent data imbalance. Each fold contained 8 to 9 P/R cases and 6 to 7 non-P/R cases. Hyperparameters were tuned to find the most robust models according to area under curve (AUC) values. Then, the best model was selected, and final performance results were obtained by repeated cross-validation. Training with a small dataset usually encounters overfitting. Therefore, random dropout layers were applied to each layer during the training process (42). Moreover, L1 and L2 regularizations were applied to fully connected layers with L1 penalty weight 1e-4 and L2 penalty weight 3e-5. The dataset is divided into training and validation sets according to 5-fold cross-validation. That is, each evaluation includes 80% data for training and 20% data for validation.

### Statistical Analysis

Statistical analyses were performed using the statistical package SPSS (V.25.0, IBM, Chicago, IL, USA). For the evaluation of clinical and radiological data, Chi-square (or Fisher’s exact test) and Mann-Whitney U tests were performed for categorical and continuous data respectively. For the evaluation of performance in DL models, the accuracy, precision, positive predictive value (PPV), negative predictive value (NPV), recall, F1 score, loss and AUC of the different prediction models were calculated. DeLong test by MedCalc statistical software (version 20.027) was used for comparison of receiver operating characteristic (ROC) curves in different DL models. Binary cross-entropy method was used for loss calculation (43). The cross-entropy loss can be calculated using the following equation:


$$\text{Binary Cross Entropy}=-\frac{1}{N}\sum_{i=1}^{N}y_{1}\cdot \log(p_{i})+(1-y_{1})\cdot \log(1-p_{i})$$

where *N* is the batch size, *p_i_* represents the predictive probability (result of the classifier) and *y_i_* represents the expected output. For all statistical analyses, *p-*values < 0.05 were considered statistically significant.

## Results

### Clinical and Radiological Features

**Figure 3:**
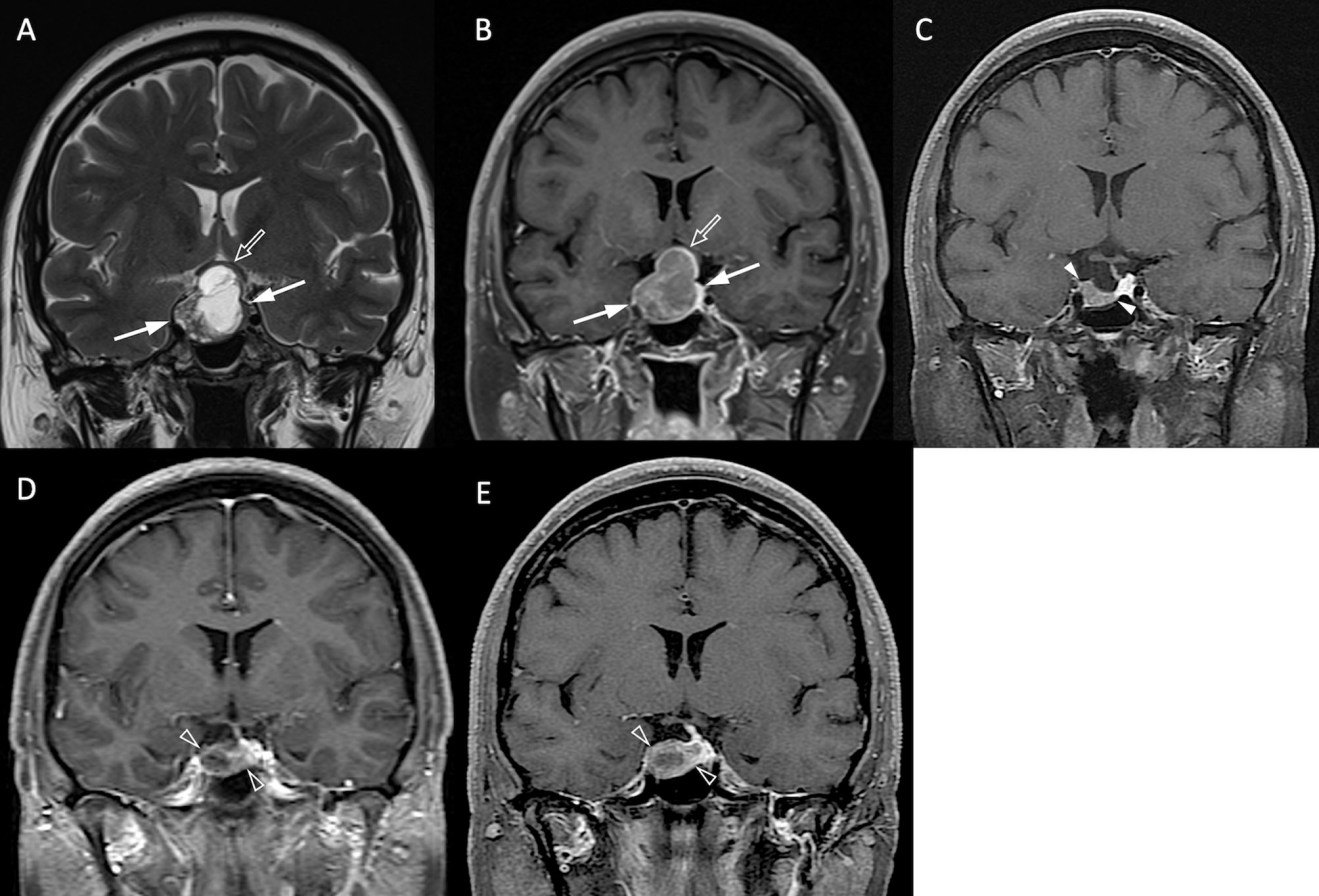
NFPA with P/R. A 45-year-old female patient with blurred vision, headache, and pathologically confirmed NFMA. **(A, B)** Coronal T2WI **(A)** and CE T1WI **(B)** show a NFMA (white arrows) with upward suprasellar extension, causing compression of the optic chiasm and the third ventricle (open arrow). **(C)** Subtotal tumor resection *via* transsphenoidal approach (TSA) was performed, and the residual tumor (arrowheads) was observed. **(D, E)** Progression of the residual tumor (open arrowheads) was observed in 27 months **(D)** and 43 months **(E)** after surgery.

**Figure 4:**
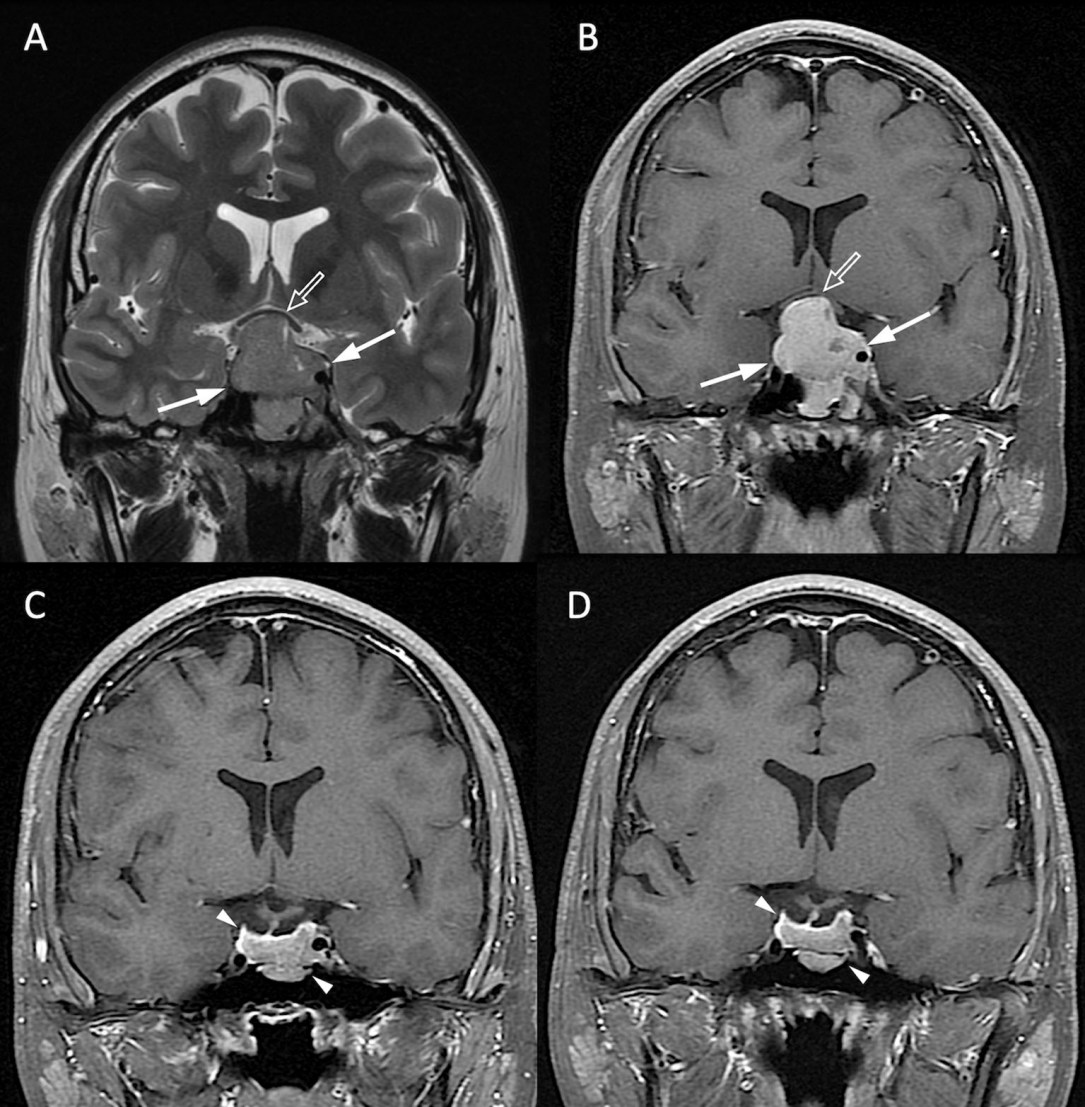
NFPA without P/R. A 20-year-old male patient with blurred vision and pathologically confirmed NFMA. **(A, B)** Coronal T2WI **(A)** and CE T1WI **(B)** show a NFMA (white arrows) with upward suprasellar extension, causing compression of the optic chiasm and the third ventricle (open arrow). **(C)** Subtotal tumor resection *via* TSA was performed, and the residual tumor (arrowheads) was observed. **(D)** No progression of the residual tumor (arrowheads) was observed 48 months after surgery.

The clinical and radiological features are summarized in **Table 1**. P/R was diagnosed in forty-two (42/78, 53.8%) patients. Among sex, age, and BMI, male sex is the most important clinical covariate in the predictive model. Significant differences (*p* < 0.05) were observed in visual disturbance, hypopituitarism, EOR, successful chiasmatic decompression, cavernous sinus/extrasellar extension, compression of the optic chiasm/3rd ventricle, and tumor height/volume between patients with and without P/R (**Figures 3**, **4**). Although significant difference in follow-up duration existed between P/R and non-P/R groups, the follow-up time in both groups (49.7 and 32 months) was more than mean time to P/R (25 months).

### Performance of CNN, MLP, and Multimodal CNN-MLP Architectures

**Figure 5:**
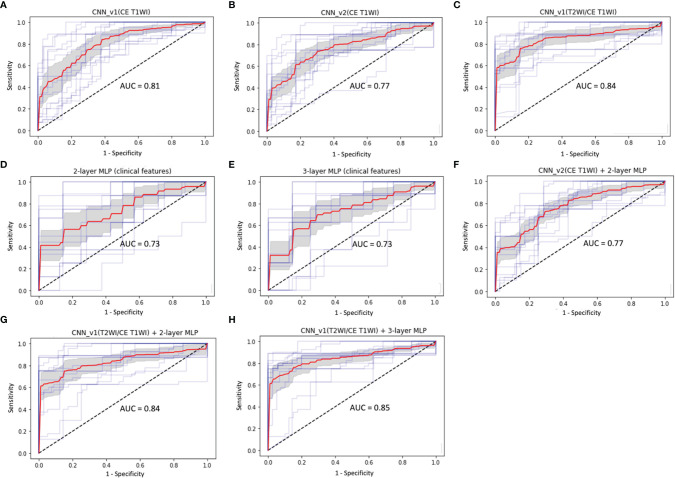
ROC curves (red: average, blue: 5 folds for cross-validation, gray: 95% confidence interval) and AUC values in **(A)** CNN_v1 (CE T1WI), **(B)** CNN_v2 (CE T1WI), **(C)** CNN_v1 (T2WI/CE T1WI), **(D)** 2-layer MLP (clinical features), **(E)** 3-layer MLP (clinical features), **(F)** multimodal CNN_v2 (CE T1WI) + 2-layer MLP, **(G)** multimodal CNN_v1 (T2WI/CE T1WI) + 2-layer MLP, and **(H)** multimodal CNN_v1 (T2WI/CE T1WI) + 3-layer MLP architectures for prediction of P/R in NFMAs.

**Figure 6:**
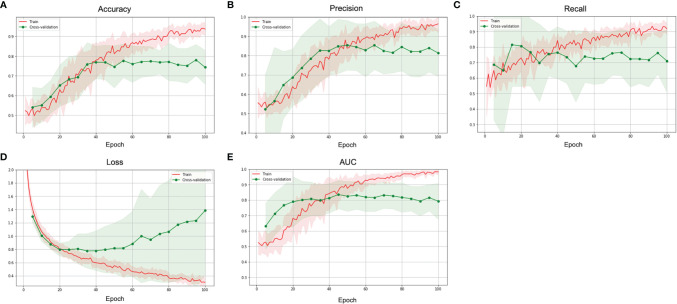
The **(A)** accuracy, **(B)** precision, **(C)** recall, **(D)** loss, and **(E)** AUC over epochs of the training (red) and validation (green) sets in the best multimodal CNN-MLP model for prediction of P/R in NFMAs.

Total 62 training cases and 16 validation cases from real patients were included. The data were extended to 6,240 training samples and 1,560 validation samples for ML. The evaluation metrics included accuracy, precision, PPV, NPV, recall, F1 score, and AUC in training and validation sets. The performance of different predictive models in the validation set are summarized in **Table 2**. All metrics were averaged using 5-fold cross validation. Among different combinations of input and model architectures, the multimodal light-weighted CNN_v1 model (using CE T1WI and T2WI) combined with 3-layer MLP (using clinical features) showed the best performance for prediction of P/R, with AUC up to 0.85 (**Figure 5**). Metrics of training and validation sets over epochs in this best predictive model are shown in **Figure 6**. In this predictive model, accuracy of 83%, precision of 90%, PPV of 89%, NPV of 78%, recall of 78%, F1 score of 0.84, and AUC of 0.85 were obtained in the validation set (**Figure 6**). **Table 3** showed comparison of ROC curves in different DL models. Although CNN_v1 model (CE T1WI and T2WI) + 3-layer MLP (clinical features) showed the best predictive performance, no statistical significance exists in AUC values between the three best predictive models: CNN_v1 (T2WI/CE T1WI) + 3-layer MLP, CNN_v1 (T2WI/CE T1WI) + 2-layer MLP, and CNN_v1 (T2WI/CE T1WI).

**Table 2:** Performance of CNN, MLP, and multimodal CNN-MLP architectures for prediction of P/R in validation set of NFMAs.

5-fold cross validation	Models	Accuracy	Precision	PPV	NPV	Recall	F1 Score	AUC
Average over 3 trials	CNN_v1(CE T1WI)	0.76	0.74	0.74	0.80	0.86	0.80	0.80
	CNN_v2(CE T1WI)	0.74	0.75	0.73	0.78	0.85	0.80	0.77
	CNN_v1(T2WI/CE T1WI)	0.83	0.87	0.86	0.79	0.81	0.84	0.84
	2-layer MLP (clinical features)	0.73	0.72	0.69	0.81	0.89	0.79	0.73
	3-layer MLP (clinical features)	0.73	0.73	0.70	0.79	0.87	0.79	0.73
	CNN_v2(CE T1WI) + 2-layer MLP	0.75	0.79	0.76	0.73	0.76	0.78	0.77
	CNN_v1(T2WI/CE T1WI) + 2-layer MLP	0.81	0.88	0.86	0.77	0.77	0.82	0.84
	CNN_v1(T2WI/CE T1WI) + 3-layer MLP	0.83	0.90	0.89	0.78	0.78	0.84	0.85

**Table 3:** Comparison between ROC curves of CNN and MLP architectures for prediction of P/R in NFMAs.

Models	CNN_v2 (CE T1WI)	CNN_v1 (T2WI/CE T1WI)	2-layer MLP (clinical)	3-layer MLP (clinical)	CNN_v2 (CE T1WI) + 2-layer MLP	CNN_v1 (T2WI/CE T1WI) + 2-layer MLP	CNN_v1 (T2WI/CE T1WI) + 3-layer MLP
**CNN_v1 (CE T1WI)**	95% CI: (-0.025, 0.028) *p* value: 0.91	(0.040, 0.097) < 0.001*	(-0.004, 0.06) 0.078	(0.015, 0.087) 0.005*	(-0.016, 0.038) 0.433	(0.029, 0.085) < 0.001*	(0.026 to 0.086) < 0.001*
**CNN_v2 (CE T1WI)**		(0.039, 0.100) < 0.001*	(-0.007, 0.066) 0.119	(0.013, 0.087) 0.008*	(-0.019, 0.038) 0.522	(0.029, 0.088) < 0.001*	(0.030, 0.085) < 0.001*
**CNN_v1 (T2WI/CE T1WI)**			(0.066 to 0.132) < 0.001*	(0.084, 0.155) < 0.001*	(0.046, 0.112) < 0.001*	(-0.007, 0.030) 0.223	(-0.010, 0.0346) 0.272
**2-layer MLP (clinical)**				(-0.014, 0.055) 0.241	(-0.015, 0.054) 0.259	(0.053, 0.122) < 0.001*	(0.050, 0.123) < 0.001*
**3-layer MLP (clinical)**					(0.002, 0.079) 0.038*	(0.073, 0.143) < 0.001*	(0.069, 0.146) < 0.001*
**CNN_v2 (CE T1WI) + 2-layer MLP**						(0.036 to 0.100) < 0.001*	(0.033, 0.100) < 0.001*
**CNN_v1 (T2WI/CE T1WI) + 2-layer MLP**							(-0.020, 0.022) 0.920

CI, confidence interval.

*Statistical difference (p < 0.05).

## Discussion

The present study explored the effectiveness of DL for prediction of tumor progression and recurrence in NFMAs. Both clinical and MRI data were used in different DL models to compare the performance between models. Several DL architectures, including CNN models using T2WI and CE T1WI data, MLP models using clinical data, and multimodal CNN-MLP models using both data were developed. Among these architectures, the multimodal CNN-MLP models using combination of clinical and MRI data showed the best performance.

Although most NFMAs (> 90%) are benign adenomas according to the 2017 WHO classification system (4), up to half of patients (25% - 55%) may exhibit early tumor P/R within 5 years after surgery (5). The Ki-67 index and cell mitosis in histopathology with tumor invasion on imaging are all associated with aggressive clinical behavior in NFMAs (4). However, the invasive growth of NFMAs is not clearly defined in the WHO criteria, and it is usually dependent on corresponding MRI study (5). For functioning pituitary adenomas, postoperative hormone concentration serves as a biomarker to detect tumor recurrence; in contrast, no specific factor is used as a marker for NFMAs (5). Conventional qualitative MR imaging features such as cavernous sinus invasion and solid tumor consistency have been reported as impact parameters associated with P/R in NFMAs (6, 8–11). Recently, low apparent diffusion coefficient (ADC) value, indicating a high cellular density, is reported to be associated with P/R in NFMAs (10, 44). However, the ADC values are often affected by susceptibility imaging artifacts from blood products due to apoplexy or necrosis in NFMAs; therefore, they can only be measured for solid tumor without hemorrhage or cystic changes (6, 10, 45). The major imaging-based ML algorithms include DL and radiomics approaches (46). As compared with conventional handcrafted radiomics, the present DL models obtain discriminative features automatically from images (47). For prediction of recurrence in NFMAs, Zhang et al. (35) first reported an accuracy of 82% and AUC of 0.78 in radiomics analysis, and superior predictive performance in DL models was obtained in the present study.

The results of clinical evaluation in NFMAs by MRI-based CNN models are excellent, and most studies report accuracy up to 90% and AUC up to 0.80 (22–30). Compared with the previously reported studies, the application of DL for predicting clinical outcomes in NFMAs have not yet been reported, and no similar studies can be compared. In our results, adding T2WI improves the predictive excellence as compared with CNN models using CE T1WI only, with AUCs of 0.84 and 0.80 respectively. For clinical features analyzed in MLP models, AUC of 0.73 in prediction of P/R can be obtained. The best performance (AUC of 0.85) can be achieved using a combination of clinical and MRI features in a multimodal CNN-MLP architecture. Herein, we have introduced this new concept concerning DL algorithms for prediction of P/R in NFMAs, although the architectures must be validated in future studies with larger sample size.

The extent of surgical resection is known to be a significant determining factor affecting tumor recurrence rates in NFMAs (8), and the present study has shown similar results. However, a significant association between the number of surgical resections and complication rates in NFMAs has been observed (48). Diabetes insipidus and anterior pituitary insufficiency are the most commonly encountered surgical complications in NFMAs, with occurrence rates of 18% and 19%, respectively (48). On the other hand, although postoperative adjuvant RT offers excellent tumor control rate in NFMAs, it may increase risks of long-term complications such as hypopituitarism, cerebrovascular accident, visual deterioration, and dementia (49, 50). Because adjuvant RT may affect the independent predictive value of the preoperative MRI-based DL analysis for P/R, patients who have received adjuvant RT before P/R were excluded from the present study. Since most NFMAs are benign tumors, preoperative prediction of tumor recurrence offers clinically valuable information for treatment options. For patients at high risks of tumor recurrence, aggressive surgical resection with adjuvant RT and close MR imaging follow-up should be considered. In contrast, for patients at lower risks of P/R, the aim of surgical treatment would be to relieve clinical symptoms by decreasing tumor mass effect. On the other hand, follow-up time is an important factor for detection of P/R in NFMAs, and it should be noticed that more recurrence may occur in patients with longer follow-up time even if the predictive model shows low risk at first. Avoiding potential surgical complications while maintaining a good treatment outcome represents optimal surgical planning for low-risk patients.

Although this is the first DL study combined clinical and MRI data for investigating tumor behavior in NFMAs, the study has several limitations. First, the retrospective study design and the limited sample size may lead to selection bias. Second, as in most imaging-based ML studies of pituitary tumors (51), the present study lacked external validation due to few available data. The MR images were acquired at a single medical center with a single protocol. Further testing with multi-institutional data and different pulse sequence protocols is necessary to determine whether the predictive model is generalizable. The inconsistency of scanning machine, magnetic field strength, and contrast agent type may affect the MR image feature. The variation in follow-up time existed between P/R and non-P/R groups due to the retrospective nature. The two-dimensional information on MR images may offer limited information to the trained model as compared with using three-dimensional convolution. Finally, when larger populations become available from more institutions, the modern CNN-based architectures such as AlexNet and GoogleNet may capture more image features, which can further improve model performance.

## Conclusions

The present study explored the effectiveness of DL in predicting P/R of the NFMAs. Even with a limited training data set, the results showed novel DL architecture incorporating clinical and MRI features provides a high level of accuracy and reliability for predicting recurrence in NFMAs. Better predictive performance was observed in a multimodal CNN-MLP model incorporating both clinical and MRI data as compared with classifiers using either clinical or MRI data alone. The results offer valuable information for preoperative and postoperative planning in NFMAs management, including the extent of surgical resection, implementation of adjuvant RT, and the time interval of MRI follow-up. Nevertheless, the DL architectures still require validation using larger-scale datasets from multiple institutions.

## Data Availability Statement

The original contributions presented in the study are included in the article/**Supplementary Material**. Further inquiries can be directed to the corresponding author.

## Ethics Statement

The studies involving human participants were reviewed and approved by Chi Mei Medical Center Institutional Review Board (IRB no. 10902-009). Written informed consent for participation was not required for this study in accordance with the national legislation and the institutional requirements.

## Author Contributions

Conceived and designed the experiments: C-CK and H-PH. Performed the experiments: Y-JC and C-CK. Analyzed the data: Y-JC, H-PH, and C-CK. Contributed reagents/materials/analysis tools: K-CH, Y-JS, and SWL. Wrote the paper: Y-JC and C-CK. Critically revised the article: Y-TK and JHC. All authors contributed to the article and approved the submitted version.

## Funding

This work was supported by the Ministry of Science and Technology (MOST) in Taiwan (MOST 109-2314-B-384-010-MY2 and MOST 110-2636-E-006-011). The funders had no role in study design, data collection and analysis, decision to publish, or preparation of the manuscript.

## Conflict of Interest

The authors declare that the research was conducted in the absence of any commercial or financial relationships that could be construed as a potential conflict of interest.

## Publisher’s Note

All claims expressed in this article are solely those of the authors and do not necessarily represent those of their affiliated organizations, or those of the publisher, the editors and the reviewers. Any product that may be evaluated in this article, or claim that may be made by its manufacturer, is not guaranteed or endorsed by the publisher.
