# Supplementary material for: Deep Learning for Prediction of Progression and Recurrence in Nonfunctioning Pituitary Macroadenomas: Combination of Clinical and MRI Features
Source: Front Oncol. 2022 Apr 20;12:813806. doi: 10.3389/fonc.2022.813806 (PMC9065347; doi:10.3389/fonc.2022.813806)
Supplement: Supplementary file 2 [file DataSheet_2.docx]

**Supplementary file 2.**

**
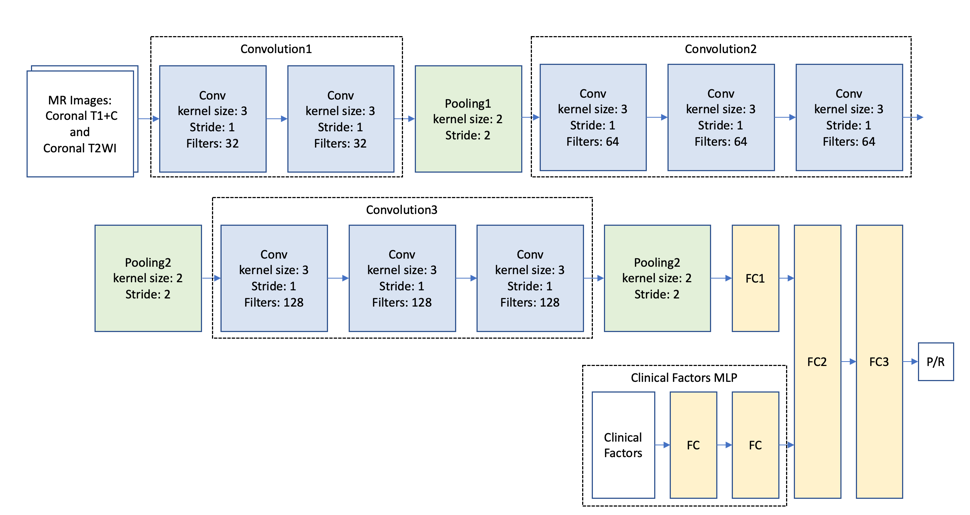
**

Figure S1. Another hybrid CNN_v2-MLP architecture for prediction of progression/recurrence (P/R) in NFMAs.
